# Mixed method evaluation of the CEBHA+ integrated knowledge translation approach: a protocol

**DOI:** 10.1186/s12961-020-00675-w

**Published:** 2021-01-18

**Authors:** Lisa M. Pfadenhauer, Tanja Grath, Peter Delobelle, Nasreen Jessani, Joerg J. Meerpohl, Anke Rohwer, Bey-Marrié Schmidt, Ingrid Toews, Ann R. Akiteng, Gertrude Chapotera, Tamara Kredo, Naomi Levitt, Seleman Ntawuyirushintege, Kerstin Sell, Eva A. Rehfuess

**Affiliations:** 1 Institute for Medical Information Processing, Biometry and Epidemiology, LMU Munich, Elisabeth-Winterhalter-Weg 6, 81377 Munich, Germany; 2 Pettenkofer School of Public Health, Munich, Germany; 3 Chronic Disease Initiative for Africa (CDIA), University of Cape Town, Groote Schuur Hospital, Observatory, Cape Town, 7925 South Africa; 4 Department of Public Health, Vrije Universiteit Brussel, Laarbeeklaan 103, Jette, 1090 Brussels, Belgium; 5 Centre for Evidence Based Health Care, Division of Epidemiology and Biostatistics, Department of Global Health, Faculty of Medicine and Health Sciences, Stellenbosch University, Francie van Zijl Drive, Parow, Cape Town, 7500 South Africa; 6 Department of International Health, Johns Hopkins Bloomberg School of Public Health, 615 North Wolfe Street, Baltimore, 21205 USA; 7 Institute for Evidence in Medicine, Medical Center - University of Freiburg, Faculty of Medicine, University of Freiburg, Breisacher Str. 86, 79110 Freiburg Im Breigau, Germany; 8 Cochrane Germany, Cochrane Germany Foundation, Berliner Allee 2, 79110 Freiburg im Breisgau, Germany; 9 Cochrane South Africa, South African Medical Research Council, Francie van Zijl Drive, Parow Valley, Tygerburg, 7500 South Africa; 10 College of Health Sciences, Makerere University, Plot 1 Upper Mulago Hill Road, Kampala, Uganda; 11 School of Public Health and Family Medicine, College of Medicine, University of Malawi, Mahatma Gandhi Road, Private Bag 360, Blantyre, Malawi; 12 Division of Clinical Pharmacology, Department of Medicine, Faculty of Medicine and Health Sciences, Stellenbosch University, Stellenbosch, South Africa; 13 University of Rwanda, KG 11 Ave Gasabo, Kigali, Rwanda

**Keywords:** Integrated knowledge translation, Co-production, Sub-Saharan Africa, Non-communicable diseases, Evaluation, Mixed methods, Comparative case study, Complexity

## Abstract

**Background:**

The Collaboration for Evidence-based Healthcare and Public Health in Africa (CEBHA+) is a research consortium concerned with the prevention, diagnosis and treatment of non-communicable diseases. CEBHA+ seeks to engage policymakers and practitioners throughout the research process in order to build lasting relationships, enhance evidence uptake, and create long-term capacity among partner institutions in Ethiopia, Malawi, Rwanda, South Africa and Uganda in collaboration with two German universities. This integrated knowledge translation (IKT) approach includes the formal development, implementation and evaluation of country specific IKT strategies.

**Methods:**

We have conceptualised the CEBHA+ IKT approach as a complex intervention in a complex system. We will employ a comparative case study (CCS) design and mixed methods to facilitate an in-depth evaluation. We will use quantitative surveys, qualitative interviews, quarterly updates, and a policy document analysis to capture the process and outcomes of IKT across the African CEBHA+ partner sites. We will conduct an early stage (early 2020) and a late-stage evaluation (early 2022), triangulate the data collected with various methods at each site and subsequently compare our findings across the five sites.

**Discussion:**

Evaluating a complex intervention such as the CEBHA+ IKT approach is complicated, even more so when undertaken across five diverse countries. Despite conceptual, methodological and practical challenges, our comparative case study addresses important evidence gaps: While involving decision-makers in the research process is gaining traction worldwide, we still know very little regarding (i) whether this approach really makes a difference to evidence uptake, (ii) the mechanisms that make IKT successful, and (iii) relevant differences across socio-cultural contexts. The evaluation described here is intended to provide relevant insights on all of these aspects, notably in countries in Sub-Saharan Africa, and is expected to contribute to the science of IKT overall.

## Background

Integrated knowledge translation (IKT) in public health and healthcare is defined as engagement of knowledge users (e.g., decision-makers) as active participants in the research process [1]. Its purpose is to engage in an interactive, collaborative process with the overarching goal being the co-production of knowledge that is relevant to policy and practice [2]. By integrating knowledge users in research, knowledge translation (KT) and research can be considered as two interwoven processes. The involved decision-makers minimise or address potential barriers that may occur when attempting to act upon results. IKT is a steadily evolving process, embedded in a complex web of contextual factors [3]. IKT thus constitutes one of a series of related concepts in the field of research co-production, namely participatory research, research collaboration, public/patient involvement and engaged scholarship, with aims ranging from the production of more applicable and useful research to the democratisation of science [4].

There are several presumed benefits linked to research that embraces IKT, the most important being that it generates knowledge that is more relevant to policy and practice and more likely to be used by decision-makers [5]. An IKT approach integrates local knowledge and helps to translate research findings into policy and practice by addressing identified knowledge-practice gaps and providing easier-to-adopt research evidence [6–8]. IKT could therefore be considered one step towards producing the right answers to the right questions [9]. The involvement of various stakeholders in health care and public health research is gaining traction [10] with increasing funder interest, predominantly in high-income countries but also in low- and middle-income countries (LMICs) [4].

While IKT shows promise in bridging the gap between research and practice, there may also be challenges encountered along the way [5]. From the perspective of decision-makers, these include presumptions about academic knowledge, skills and capabilities [5]. Functional collaborations require time to be established and effort to be maintained, resources that are often not allocated by funders [11, 12]. Stakeholders may have competing demands and agendas [11] and may consider a range of aspects before engaging in IKT [2]. Also, networks relying on personal relationships might be affected by personnel changes [11, 13–15] as well as the “maturity” of the relationships [8, 16]. Last but not least, it should be acknowledged that IKT is not a panacea and is unlikely to be a suitable approach for all research endeavours [11]. Thus it is important to gauge where and when an IKT approach adds value to research.

Considering that IKT has been assumed to have an influential role in evidence-informed policy-making, it is surprising that high-quality evaluations of its processes and outcomes are still scarce [17]. Most evaluation research has been conducted in the form of case studies, using observational study designs [18]. A scoping review concluded that IKT initiatives that were evaluated achieved one or more positive outcomes, however, due to the often poor quality and reporting of these evaluations, no recommendation with regards to what makes IKT strategies effective or not could be made [19]. The IKT field should therefore emphasise a thorough description of IKT efforts and their implementation, as well as well-conducted evaluations [20]. Also, formative research to develop and test outcome measures such as metrics of research use or research impact on policy making would help to advance the knowledge translation field [20]. The field needs empirical and comparative research that tests the benefits that are attributed to IKT, such as the generation of social capital, new relations and cooperative behaviour, greater quality of health services and reduced costs and greater health benefits for those involved [21, 22], including an increased consideration of contextual factors [23]. Also, LMICs are seldom represented in IKT evaluations, with evidence to date derived from a small number of primarily Anglophone countries, notably the United States, United Kingdom, Canada, Australia and New Zealand [21, 23, 24].

IKT represents an integral part of the *Collaboration for Evidence-Based Healthcare and Public Health in Africa* (CEBHA+), an African-German research consortium with a focus on non-communicable diseases (research tasks 1—3: prevention, screening and treatment of cardiovascular disease, diabetes, and hypertension; research task 4: road traffic injuries). CEBHA+ is a five-year project funded by the German Federal Ministry of Education and Research (BMBF) as part of the Research Networks for Health Innovation in Sub-Saharan Africa funding initiative. It was set up in 2017 and aims to build long-term capacity and infrastructure for evidence-based health care and public health in Africa. The consortium consists of seven partner institutions in five Sub-Saharan African countries (Ethiopia, Malawi, Rwanda, Uganda, and South Africa; subsequently referred to as five “CEBHA+ sites”) and two partner institutions in Germany. As one of its core goals, CEBHA+ seeks to promote the use of contextualised research evidence in decision-making by applying a coordinated IKT approach across all its research activities. To this end, researchers at the five African CEBHA+ sites are pursuing focused, long-term engagement with members of the policy-and-practice community (subsequently referred to as “stakeholders”).

## Objective

This paper describes the evaluation procedures to assess the process and outcomes of the CEBHA+ IKT approach implemented across five African CEBHA+ sites. The evaluation is based on a programme theory and will be conducted semi-externally, i.e. one of the two German partner institutions, the LMU Munich (“the evaluation team”), will lead the evaluation while not being directly involved in the implementation of the IKT strategies at each site.

The proposed outcome evaluation aims to assess whether an IKT approach ultimately contributes to increased uptake of contextualised research in policy and practice, while also paying attention to intermediate outcomes such as increased relevance or applicability of research in the respective context. The proposed process evaluation, conducted in parallel, aims to shed light on the dose, fidelity and quality of the IKT strategies implemented at each site [25]. Continuous monitoring, conducted by each implementing African partner site, is envisioned to refine the IKT strategies as they are being implemented.

Having conceptualised the CEBHA+ IKT approach as a complex intervention in a complex system, our evaluation is expected to support an in-depth understanding of what works, for whom, and in which circumstances. We aim to enhance our understanding of IKT within the CEBHA+ consortium and beyond; to enhance the IKT approach over the course of the project; and, to contribute to the IKT research field as a whole.

## Methods

### Intervention: overarching CEBHA+ IKT approach and site-specific IKT strategies

The IKT approach in CEBHA+ can be conceptualized as an intervention within a complex social system. By implementing a systematic approach to engaging with stakeholders, building new or strengthening existing relationships, we aim to disrupt traditional knowledge translation approaches [26]. We do this by redistributing and transforming resources, for example by having dedicated personnel for IKT (“IKT focal points”, one or two staff per site) that coordinate and implement local IKT efforts. The IKT focal points and the IKT evaluation team at LMU Munich make up the CEBHA+ IKT team.

At each CEBHA+ site, researchers engage with stakeholders. In the context of IKT in healthcare and public health, a concerned stakeholder can be defined as an entity responsible, involved or affected by health-related decisions that can be informed by research evidence [27, 28]. Entities can be individuals, organisations, groups or networks operating at a local, regional, national or supra-national level. They can comprise the public, civil society organizations, the media, public health practitioners and service providers as well as public health policy-makers and industry actors across multiple sectors [29]. These entities can be directly or indirectly affected—in positive or negative ways—by an effort or the actions of an entity [30]. We target decision-makers, i.e. policy and practice stakeholders holding a position that allows them to make system-level, organizational or technical decisions that affect the general health of communities or populations. These decisions can be made on a more political or a more technical (i.e. programmatic) level within the health sector as well as in other sectors. Our primarily targeted stakeholder group might be extended as we proceed with the CEBHA+ IKT approach.

Initially, the IKT evaluation team at LMU Munich conducted a literature review on IKT, its mechanisms and effectiveness. We conducted forward citation tracking from a recent scoping review [19]. This search (Feb 2018) did not yield updates of the review or more recently published evaluations of IKT approaches. Informed by the literature review and discussions with the IKT focal points, we developed a programme theory of the overarching CEBHA+ IKT approach. This programme theory was operationalized into a menu of options of IKT activities that researchers and stakeholders could adapt to their respective research tasks and contexts.

**Fig. 1:**
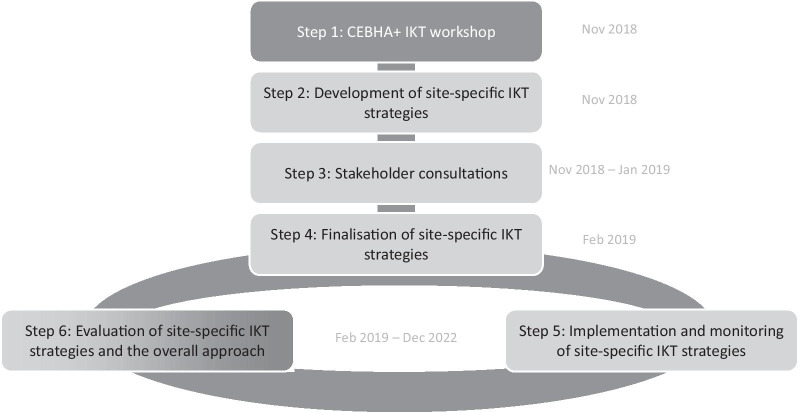
Development, implementation and evaluation of integrated knowledge translation approach in CEBHA+. This figure visualizes the process of developing, implementing and evaluating the integrated knowledge translation approach in CEBHA+. The dark shaded boxes describe steps which were pursued at the level of the research consortium, while the light shaded boxes describe steps pursued at the respective sites. Step 6 (evaluation) is pursued at both the consortium and individual site level

The overall CEBHA+ IKT approach comprises six steps, of which four are site-specific and include development of IKT strategies that are tailored to each CEBHA+ site. These site-specific IKT strategies recognise existing relationships at the different institutions, the local context, and the varying needs of different research tasks within CEBHA+. A coordinated IKT approach ensures that site-specific IKT strategies are harmonised to the extent reasonable. In terms of activities, the six steps towards a coordinated IKT approach in CEBHA+ are described in Table 1 and visualized in Fig. 1.

**Table 1:** Overview of development, implementation and evaluation of the integrated knowledge translation approach in CEBHA+

Step	Date	Description
Step 1	Nov 2018	In a foundational workshop developed and implemented by the Centre for Evidence Based Health Care, Stellenbosch University [31], one of the South African CEBHA+ partners, IKT focal points from each CEBHA+ site were introduced to the concepts of evidence-informed decision-making, IKT, and the overarching CEBHA+ IKT approach. Participants were introduced to the programme theory and received practical training on how to develop an IKT strategy (stakeholder mapping, stakeholder analysis, stakeholder engagement strategies) and draft a site-specific IKT strategy
Step 2	Nov 2018	Workshop participants acted as multipliers within their country teams and shared their knowledge and skills gained during the workshop with the rest of their team members. By using the tools introduced during the workshop, country teams were requested to jointly agree on priority stakeholders with whom to engage over the course of the CEBHA+ project and to refine the site-specific IKT strategy drafted during the foundational workshop
Step 3	Nov 2018 –Jan 2019	Each country team consulted priority stakeholders to introduce CEBHA+and IKT and to gauge their interest in the research topic as well as their preferences for engaging with the CEBHA+ research team
Step 4	Feb 2019	Country teams met to review and synthesise the information obtained in previous steps and to finalise the site-specific stakeholder analysis and IKT strategy
Step 5	Feb 2019–Dec 2022	Country teams are implementing and refining their site-specific IKT strategies and monitoring the indicators they defined as part of their site-specific IKT strategy
Step 6	Feb 2020–Jul 2022	A two-step evaluation, by LMU Munich, of the coordinated IKT approach and the site-specific IKT strategies will assess the added value of the IKT approach in CEBHA+ (stage one ongoing, stage two scheduled for Mid-2022)

These steps are not necessarily pursued in a linear manner across all sites but require a certain degree of iteration, i.e. steps 2 to 5 may be repeated as the stakeholder landscape changes. Ideally, stakeholders are engaged in the research process from an early stage [11]. This engagement can comprise shaping the research questions, deciding on the methodology, involvement in data collection and development of tools, interpreting the findings and supporting dissemination of research results at the research task level [2]. At the level of the CEBHA+ consortium, this “IKT thinking” has been embraced since the initial development of the project proposal, where relevant stakeholders from the respective partner countries were included in the process of setting research priorities. Over the course of the project, practical experiences with the development and implementation of site-specific IKT strategies, insights gained from monitoring and evaluation, or new scientific evidence may lead to adaptations in the overall IKT approach and/or the site-specific IKT strategies.

### Programme theory

**Fig. 2:**
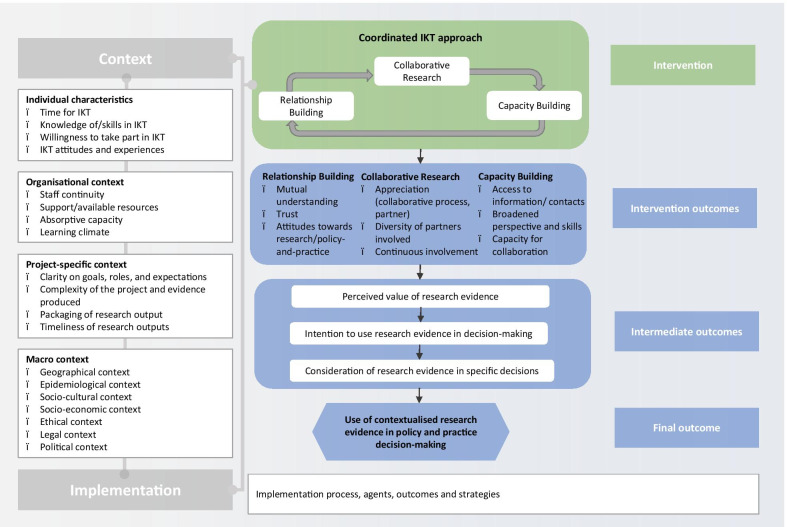
Programme theory of integrated knowledge translation approach in CEBHA+

In order to guide the development and evaluation of the CEBHA+ IKT approach, we developed a comprehensive programme theory (see Fig. 2). This builds upon two main frameworks: the Context and Implementation of Complex Interventions (CICI) framework [32] and a visual representation of IKT approaches, influencing factors, and outcomes as described in a scoping review by Gagliardi et al. [19]. The CICI framework was used to structure the context and implementation component of the evaluation by providing a priori categories which informed the development of both the qualitative interview guide and the survey. The framework by Gagliardi provides an overview of empirical evaluations of IKT approaches, relevant enablers and barriers, preconditions, and outcomes. Moreover, it flags relevant and partly validated evaluation tools which informed the development of our survey. The contributions of these two frameworks were integrated to show how the CEBHA+ IKT approach is assumed to increase the use of contextualized research evidence in policy and practice decision-making.

As shown in Fig. 2, the programme theory describes the coordinated IKT approach as an *intervention* implemented across the CEBHA+ consortium. In CEBHA+, a coordinated IKT approach is understood as an iterative process in which (1) relationship building and strengthening, (2) capacity building, and (3) collaborative research between CEBHA+ researchers and their priority stakeholders represent integral components of the intervention. Capacity building, relationship building, and their maintenance and collaborative research are expected to take place in an interactive manner and repeatedly over time, thereby reinforcing each other [3].

The implementation of these intervention components is expected to result in several *intervention outcomes.* The literature indicates that strong relationships increase mutual understanding (e.g. of language, work style, needs, constraints) and trust among researchers and decision-makers engaging in a partnership and that these influence attitudes towards the partners’ professional environment (i.e. attitudes towards research among stakeholders versus attitudes towards the policy-and-practice field among researchers) [19, 33–36]. With this in mind, CEBHA+ researchers seek to build and enhance relationships with priority stakeholders. The experience of conducting collaborative research would ideally lead to appreciation for the collaborative process [33], promote a more diverse range of partners to engage in, and encourage involvement throughout the research process. Mutual capacity-building can occur in the form of improved access to information and/or contacts due to the research partnership, broadened perspectives and skills (e.g. research skills), and improved capacity for collaboration [33–35].

Moving on to *intermediate outcomes*, successful research partnerships between researchers and stakeholders is assumed to result in an increased perceived value of research evidence in terms of relevance, better applicability to the stakeholders’ field of activity as well as credibility. While both actual and perceived value are potentially affected by IKT efforts, studies show the individual perception of value has greater behavioural implications than the actual value of research evidence [37]. We therefore focus on the perceived value of CEBHA+ research evidence with respect to its importance to the actual consideration and utilization of research evidence in decision-making. In turn, research evidence that is perceived as credible, relevant, and usable is likely to result in an increased intention to use that evidence in policy and practice decision-making. Subsequently, the intention to use research evidence is hypothesised to lead to the actual consideration of findings in a specific decision in another step towards the *final outcome:* the use of contextualized research in policy and practice decision-making.

### Evaluation of the CEBHA+ IKT approach

#### Overview of evaluation approach

This evaluation is theory-driven. The programme theory provides the main structure for the evaluation and informs our choice of methods for data collection as well as data analysis. A comparative case study (CCS) design [38] will be used, including mixed methods to facilitate in-depth examination of IKT evaluation in each site. Each site (Ethiopia, Malawi, Rwanda, South Africa, Uganda) is considered a case. A CCS is undertaken over time and emphasizes comparison within and across contexts and thus allows to explore, understand, and explain how context influences the success of an intervention [39]. Studying each case with its distinct IKT strategy at two time points will provide an opportunity to compare and contrast findings within and across sites.

As part of the process evaluation, we will look at how and why IKT works in the respective contexts. In line with the CICI framework, we will look at the different stages in the implementation process, the diverse implementation strategies employed in different sites and the respective implementation agents [32]. We will moreover utilize our monitoring to deepen our understanding of the implementation process. This may be complemented by including quantitative implementation outcomes, such as acceptability and feasibility in the late-stage evaluation. As part of the outcome evaluation, we will investigate the intervention, intermediate, and final outcomes, as defined in the programme theory.

**Fig. 3:**
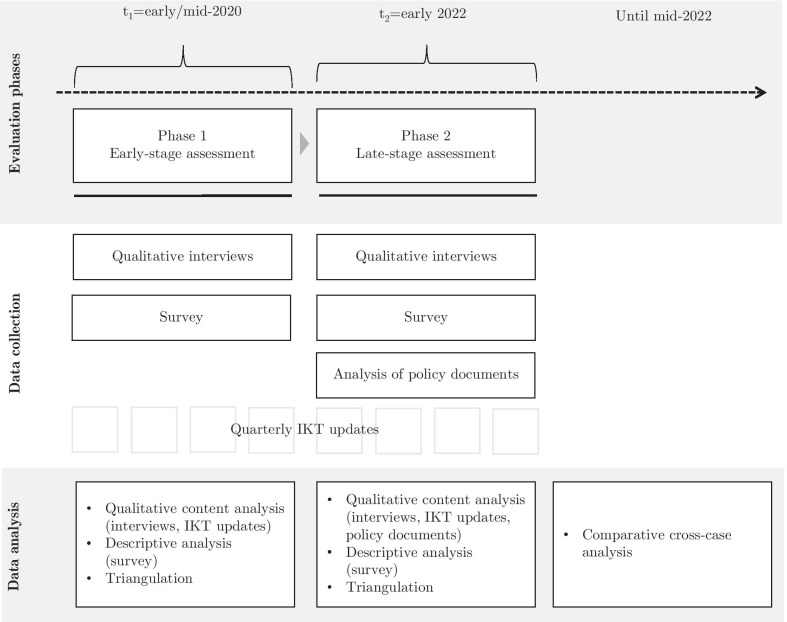
Overview of evaluation of integrated knowledge translation approach in CEBHA+

As shown in Fig. 3, the evaluation process will comprise two phases, i.e. an early-stage assessment (early 2020) and a late-stage assessment (early 2022). In addition to the data analyses performed at the end of each phase, we will conduct an integrated analysis after the completion of phases 1 and 2 (until mid-2022). The evaluation process will comprise five different procedures: (a) quantitative surveys with all CEBHA+ priority stakeholders as well as CEBHA+ researchers; (b) qualitative interviews with all CEBHA+ priority stakeholders as well as CEBHA+ researchers; (c) continuous monitoring to capture ongoing IKT activities (mainly covered by the documentation of interactions between CEBHA+ researchers and policy-and-practice stakeholders reported in quarterly updates); (d) policy document analysis; and (e) integrated and cross-case analysis at the end of the evaluation. Each of the procedures will be conducted in all five CEBHA+ sites.

#### Data collection

Given the complexity of the initial programme theory and the multitude of outcomes along the causal path from the intervention to the intended outcome, we will use a combination of qualitative (semi-structured interviews, qualitative analysis of policy documents, IKT updates) and quantitative data collection approaches (survey). The survey is intended to touch upon all dimensions covered by the programme theory. The qualitative interviews, however, will allow us to explore how IKT exerts an influence in more depth. Also, we will focus on external context factors (macro-context) that impact on IKT, the process of IKT, as well as intermediate and final outcome(s). Two distinct surveys and two interview guides target researchers and stakeholders, respectively. This is necessary to reflect the different perspectives of individuals engaged in a partnership.

#### Development of data collection instruments

The underlying programme theory was used to develop survey and interview instruments. We initially constructed the survey based on suitable pre-existing instruments included in the review by Gagliardi et al. (2015). Literature searches were then conducted and instruments identified to inform the development of additional survey items (e.g. SAGE [40], PreVAiL [41], SEER [42]). Where items were non-existent, we constructed them de novo. The survey comprises both multiple choice and Likert scale questions. We provide an overview of included constructs and sub-constructs as well as their source in the additional files (Additional file 1).

The interview guides and surveys were pilot tested among two researchers (Rwanda and South Africa, one male, one female) who are not involved in the IKT implementation process. We assessed the tools for errors, comprehensibility, acceptability, and understandability. We were not able to pilot-test our tools with decision-makers from policy-and-practice due to their limited time availability and the high opportunity cost. Both the interview guide and survey were slightly adapted to account for the changed project context due to the COVID-19 pandemic and are available as additional files (Additional file 2, Additional file 3, Additional file 4, Additional file 5).

Insights gained during the early-stage evaluation will inform the interview guides and surveys used in the late-stage evaluation. In addition to the topics covered in the early-stage assessment, participants will also be asked to describe how the research partnership has changed over time and to describe, where applicable, examples of successful translation of research evidence into policy or practice because of the research partnership.

#### Sampling

Selection of study participants for the surveys and interviews will reflect the following criteria:

- A CEBHA+ researcher or a priority stakeholder as identified by the respective CEBHA + country team; AND
- actively involved in a research-stakeholder partnership in CEBHA+ ; AND
- 18 years or above; AND
- lives and works in the respective CEBHA+ country; AND
- is able to understand and articulate him/herself in English

With respect to sample size, we aim to interview five to ten participants per site, ideally both researchers and key stakeholders.

#### Recruitment

The evaluators will invite all eligible CEBHA+ researchers to participate in the study via email and will introduce the study. Eligible stakeholders will be invited to participate by their respective research counterparts and will be provided with the same information.

Participants willing to be enrolled will be contacted by the evaluators via email to arrange a date for the qualitative interview. A written informed consent form will be provided, detailing the study’s goals, estimated duration, scope, its voluntary nature, the pseudonymity and confidentiality of responses, and whom to contact regarding questions. Participants will be asked to return the digitally signed informed consent form or a photo/scan of the manually signed form. Participants will also receive an invitation link to access the online survey.

#### Survey procedures

The survey will be administered online using the LimeSurvey tool (https://www.limesurvey.org/en) and participants requested to complete the survey prior to the qualitative interviews. At the beginning of the questionnaire, participants will receive information regarding the study. Key information regarding the informed consent form will be reiterated. Participants who are not able to complete the survey before the qualitative interviews will receive reminders via email and orally during the interview.

#### Interview procedures

The interviews will be conducted by a member of the CEBHA+ evaluation team or trained research staff at the CEBHA+ site using an interview guide. Where feasible, notes will be taken by a second member of the CEBHA+ evaluation team or by trained research staff at the CEBHA+ site, who are not directly involved with the site-specific IKT strategy. We consider it important that the interviewer is not involved in the practical implementation of IKT at the respective CEBHA+ site and thus unknown to the interviewed stakeholder to reduce interviewer bias. All interviews will be conducted in English.

In light of the COVID-19 pandemic interviews will be conducted virtually (e.g. via skype, zoom or other) unless the interviewee insists on a face-to-face interview, the pandemic situation permits, and precautionary measures are in place. Where conducted in person, interviews will be carried out in a place of the interviewee’s choice, e.g. the office of the stakeholder or researcher. As a minimum requirement, the chosen place should be a separate room or another quiet place to avoid disturbances and outside observers. The estimated duration of a single interview is 30 to 60 min.

The interviewees will be asked to describe their perspective on the research partnership, contextual factors that hinder or facilitate the partnership or its outcomes, the expected benefits and ways to improve the partnership and/or outcomes. Interviews will be audio-recorded and transcribed verbatim. Interviewees will be given the opportunity to review the transcript, ask for modifications and give their approval for further use of the data.

#### Further data sources

Quarterly IKT updates serve as a means of continuous exchange among the IKT teams. The updates take place during a virtual call and are facilitated by a structured reporting tool. This tool captures noteworthy interactions between researchers and stakeholders and their outputs. It also contains questions on the learnings and challenges encountered at each site. All updates are submitted to the evaluation team before the calls and will be analysed in Phase 1 and 2.

Policy documents will be identified and analysed for the late-stage assessment. Relevant policy documents are those produced between 2017 and 2022 (i.e. the course of the CEBHA+ project) and on topics related to CEBHA+ research activities at the five research sites. Documents concerned with laws, regulations, plans and major programmes from different tiers of government will be selected. Other documents considered will include those issued by multi-sectoral working groups or semi-independent bodies with a mandate to devise strategies or plans. These policy documents will be identified through the quarterly IKT updates, interviews and stakeholder websites (incl. meeting agendas, minutes, protocols, or reports).

### Data analysis

#### Descriptive analysis of survey data

All quantitative data will be analysed descriptively using IBM SPSS Statistics 27.0. Survey data will be analysed in conjunction with qualitative data for triangulation at the end of phase 1 and phase 2 (Fig. 3).

#### Qualitative analysis of interviews

Interview transcripts will be analysed in MaxQDA using content analysis as described by Schreier [43]. Codes will be developed both deductively (informed by the programme theory and resulting interview guide) and inductively (data driven).

For each of the sites, the interviews will be coded independently by two coders, with one of them being from the same site with a comparable cultural background as the interviewee in order to include both an insider as well as outsider perspective [44]. Codes will be discussed among the two coders and adapted until agreement is reached. For each site, there will be two sets of category systems, one for the researcher and one for the stakeholder dataset. While we expect overlaps, there are likely to be distinctions. This will ultimately lead to a code book that will be applied to all interviews, while allowing for inductively developed, site-specific codes.

#### Document analysis of policy documents

The policy documents identified in the late-stage evaluation phase will be analysed through document analysis, a systematic procedure for reviewing or evaluating printed or electronic documents, which entails finding, selecting, appraising and synthesising data contained in the retrieved documents [45]. We will look for specific indicators of CEBHA+-generated research evidence uptake into practice or policymaking. Qualitative content analysis will be applied to relevant sections of the document [45], by using an a priori developed list of categories which corresponds to the outcome indicators in our programme theory. Text passages that discuss any of these a priori categories will be coded according to this list, while others will be coded under new categories. The resulting category systems will be shared and discussed among the CEBHA+ IKT team members undertaking the document analyses.

#### Triangulation of data at each site

At the end of phases 1 and 2, qualitative and quantitative data will be integrated for each case using the triangulation method as described by Moran-Ellis and colleagues (2006), “following a thread” [46]. Triangulation is a general approach whereby the convergence, complementarity, and dissonance of results on related research questions, obtained through different methodological approaches, sources, theoretical perspective, or researchers are explored. With this method, an initial analysis of multiple datasets (survey, interviews, quarterly IKT updates, policy document analysis) will be undertaken separately for each case (i.e. each site). At this stage, themes and analytical questions that require further investigation will be identified. These themes or questions—the thread—are followed throughout the datasets to capture corresponding, contradicting as well as lacking findings *within cases.* The final outcome of this step is to create concise case descriptions for each of the five individual CEBHA + sites.

#### Cross-case analysis across sites

After data collection and triangulation (2022), we will conduct a cross-case analysis across findings from the five CEBHA+ sites, the two points of data collection and various methods of data collection. Cross-case analysis is a research method that facilitates the comparison of commonalities and differences in the events, activities, and processes that are the units of analyses in case studies. The aim is to compare key findings *across cases* (i.e. across sites). Drawing upon the programme theory, we will contrast what we found at each site with regards to the components of the programme theory. This cross-case analysis will also serve as basis for reflections on the community of IKT practice across sites within the CEBHA+ consortium. Insights from the cross-case analysis are likely to lead to refinements of the initial programme theory, leading to a middle-range theory.

#### Data management

Data collection, retention and analysis will be conducted adhering to EU data protection regulations. All data (digital records, transcripts, interview notes, survey data) will be kept confidentially on a secured institutional IT infrastructure provided by the Institute for Medical Information Processing, Biometry, and Epidemiology (IBE) at the LMU Munich, which meets the requirements of an institute for research on highly sensitive patient and study data. For analysis, researchers from the respective CEBHA+ sites will have access to the pseudonymized interview data after transcription. All analyses will be undertaken in the cloud and no data will be stored locally at any of the sites.

## Discussion

To our knowledge, this protocol is one of the first attempts to evaluate an IKT approach across multiple sites and countries using a longitudinal design. It suggests an overall methodology and detailed methods which we deem appropriate to capture the complexities and context dependencies of the different IKT strategies employed across sites and highlight the important characteristics of individuals involved in delivering IKT. The opportunity that a thorough development, implementation and evaluation of IKT is possible within the context of CEBHA+ can be attributed to the open funding call issued by the German Federal Ministry of Education and Research (*Bundesministerium für Bildung und Forschung*, BMBF) in 2013. Whilst the funder’s interest in IKT as a means of enhancing research impact is highly commendable and in line with several other international funding agencies requiring that knowledge translation be integrated into proposals [12], a critical meta-discussion is needed on how IKT interventions can be aligned with the realities of international research collaborations and the contexts and capacities of the different partner institutions.

We have encountered and will continue to encounter conceptual, methodological and practical challenges in the proposed evaluation. Conceptually, we are seeking to understand the context and individual dependency of IKT which will inevitably lead to heterogeneity. We aim to capture these heterogeneities by employing multiple methods; we endeavour to paint a holistic picture by integrating the perspectives of researchers and stakeholders in the evaluation.

Methodologically, we have struggled to identify appropriate and valid ways of monitoring highly heterogeneous IKT strategies. This will be particularly important when determining the dose–response-relationship between the effort invested in stakeholder engagement and related outcomes. We are optimistic that the triangulation of different sources of data will provide valuable insights. We have conceptualized this evaluation as semi-external, with the evaluation team not being involved in the implementation of local IKT strategies. While this more distant perspective on the realities of IKT on the ground facilitates a largely independent evaluation, the interpretation of evaluation findings will need to be complemented by local researchers’ contextual understanding. This will be facilitated by integrating one researcher per site with the analysis and interpretation of the generated data. This researcher will not have participated in the evaluation.

Practically, CEBHA+ activities just like those of most other health research institutions or networks have been severely disrupted by the COVID-19 pandemic. Both on the side of the researchers and the stakeholders, resources initially intended for CEBHA+ activities were focused towards responding to emerging needs due to the pandemic. Additionally, we had to defer from conducting this evaluation face-to-face and resort to virtual interviews. These developments led to delays and reiterations in our procedures. On the other hand, the COVID-19 pandemic has most clearly demonstrated the international need for research evidence in practice and policy decision making and made a very strong case for IKT. While we cannot anticipate the developments over the next year(s), we believe that the IKT experiences in CEBHA+ thus far do contribute to the international research on IKT, especially given the intensive collaboration of researchers and policymakers during the pandemic.

## Conclusion

While involving decision-makers in the research process is gaining traction worldwide, there is still very little knowledge regarding (i) whether a systematic approach makes a difference to evidence uptake, (ii) the mechanisms that make IKT successful, and (iii) relevant differences across socio-cultural contexts. The CEBHA+ IKT approach represents a carefully developed intervention designed to increase the use of contextualized research in policy and practice decision-making. As an approach that is implemented in a coordinated manner across five African countries, it represents a rare opportunity for a thorough evaluation and learning from differences across multiple sites. The evaluation described here is intended to provide relevant insights on all the above aspects, notably in Sub-Saharan Africa, and is expected to contribute to the science of IKT overall.

## Supplementary Information

**Additional file 1:** Evaluation domains, constructs and data sources

**Additional file 2:** CEBHA+ evaluation survey for researchers

**Additional file 3:** CEBHA+ evaluation survey for stakeholders

**Additional file 4:** CEBHA+ evaluation interview guide for researchers

**Additional file 5:** CEBHA+ evaluation interview guide for stakeholders

## Data Availability

Not applicable.

## Abbreviations

IKT/KT: Integrated knowledge translation/knowledge translation
CEBHA+: Collaboration for Evidence-Based Healthcare and Public Health in Africa
CCS: Comparative case study
LMIC: Low- and middle-income country
