# Supplementary material for: Mixed method evaluation of the CEBHA+ integrated knowledge translation approach: a protocol
Source: Health Res Policy Syst. 2021 Jan 18;19:7. doi: 10.1186/s12961-020-00675-w (PMC7813167; doi:10.1186/s12961-020-00675-w)
Supplement: Supplementary file 1 — Additional file 1: Evaluation domains, constructs and data sources [file 12961_2020_675_MOESM1_ESM.docx]

# Additional File 1: Evaluation Domains, Constructs and Data Sources

| Domain | Construct | Subconstruct | Source survey construct | CEBHA+ tool |
| --- | --- | --- | --- | --- |
| Intervention | Relationship building | See below under “intervention outcomes” |  |  |
|  | Capacity building | - IKT capacity (stakeholder + CEBHA+ researcher) - Capacity to collaborate with researchers, respectively policy and practice stakeholders in a mutually beneficial research project/ programme. - Research capacity (stakeholder) | (1) | Survey  Interview |
|  | Collaborative research | - Appreciation for the collaborative process and partner - Diversity of partners involved - Continuous involvement | (2, 3) | Survey  Interview |
| Intervention Outcomes | Capacity for collaboration |  | (1, 3) | Survey |
|  | Broadened perspective and skills | - Perspective - Research skill | (4, 5) | Survey |
|  | Access to information/ contacts | - Information about other pertinent research - New contacts with other researchers or decision-makers | (4, 6) | Survey |
|  | Attitudes towards research/ policy and practice | - Relevance of research - Value of research - Credibility of research | (7) | Survey |
|  | Appreciation | - Appreciation for the partner - Appreciation for the collaborative process | (2, 3) | Survey |
|  | Mutual understanding | - Work style - Language - Needs/ constraints | (2, 3) | Survey |
|  | Diversity of partners involved |  | (3) | Survey |
|  | Continuous involvement |  | (2) |  |
|  | Trust | Trust and Mistrust in partners | (8) | Survey |
| Intermediate Outcomes | Perceived value of research evidence | - Individual view on relevance of CEBHA+ research output - Individual view on applicability/ positioning for use - Individual view on credibility | Developed de novo | Survey  Interview |
|  | Intention to use research evidence in decision-making | - Behavioural intention - Attitudes - Subjective norms - Perceived behavioural control | (9) | Survey  Interview |
|  | Consideration of CEBHA+ research evidence in daily work | Ways of use of research evidence   - Conceptual - Instrumental - Tactical/ symbolic - Imposed | (5, 10) | Survey |
| Final Outcomes | Use of contextualised research evidence in policy and practice decision-making |  | Developed de novo | Survey  Documents |
| Context | Macro context | - Geographical context - Legal context - Ethical context - Socio-Cultural context - Socio-Economic context - Political context - Epidemiological context | (11) | Interview  Documents  Monitoring |
|  | Organisational context (Meso Context) | - Staff continuity - Organisational support/ available resources - Organisational absorptive capacity | (2)  (12) | Survey  Interview |
|  | Project-specific context | - Clarity on goals, roles, and expectations - Complexity of the project and evidence produced - Packaging of research outputs - Timeliness of research outputs | (2)  Developed de novo  (2)  (13) | Survey  Interview |
| Implementation | Individual characteristics of the implementation agent | - Time for IKT - Knowledge of/ skills in IKT - Willingness to take part in IKT - IKT attitudes and experiences | Developed de novo  (12) | Survey  Interview |
|  | Implementation strategies |  | (11) | Interview  Monitoring |
|  | Implementation process | - Decision to adopt - Planning and preparation: Initial implementation - Full implementation Evaluation and reflection - Sustainment | (11) | Interview |

# References

- 1. Abelson J, Li K, Wilson G, Shields K, Schneider C, Boesveld S. Supporting quality public and patient engagement in health system organizations: development and usability testing of the Public and Patient Engagement Evaluation Tool. Health expectations : an international journal of public participation in health care and health policy. 2016;19(4):817-27.
- 2. Kothari A, Sibbald SL, Wathen CN. Evaluation of partnerships in a transnational family violence prevention network using an integrated knowledge translation and exchange model: a mixed methods study. Health Res Policy Syst. 2014;12:25.
- 3. Abelson J, Li K, Wilson G, Shields K, Schneider C, Boesveld S. Supporting quality public and patient engagement in health system organizations: development and usability testing of the Public and Patient Engagement Evaluation Tool. Health Expectations. 2016;19(4):817-27.
- 4. King G, Servais M, Kertoy M, Specht J, Currie M, Rosenbaum P, et al. A measure of community members' perceptions of the impacts of research partnerships in health and social services. Evaluation and program planning. 2009;32(3):289-99.
- 5. Brennan SE, McKenzie JE, Turner T, Redman S, Makkar S, Williamson A, et al. Development and validation of SEER (Seeking, Engaging with and Evaluating Research): a measure of policymakers' capacity to engage with and use research. Health research policy and systems. 2017;15(1):1-.
- 6. Oliver KA, de Vocht F, Money A, Everett M. Identifying public health policymakers’ sources of information: comparing survey and network analyses. European Journal of Public Health. 2015;27(suppl_2):118-23.
- 7. National Center for Research in Policy and Practice (NCRPP). Survey of Practitioners’ Use of Research 2016 [Available from: <http://ncrpp.org/assets/documents/NCRPP_SPUR-Instrument.pdf>.
- 8. Jones J, Barry MM. Developing a scale to measure synergy in health promotion partnerships. Global health promotion. 2011;18(2):36-44.
- 9. Boyko JA, Lavis JN, Dobbins M, Souza NM. Reliability of a tool for measuring theory of planned behaviour constructs for use in evaluating research use in policymaking. Health Research Policy and Systems. 2011;9(1):29.
- 10. Makkar SR, Brennan S, Turner T, Williamson A, Redman S, Green S. The development of SAGE: A tool to evaluate how policymakers’ engage with and use research in health policymaking. Research Evaluation. 2016;25(3):315-28.
- 11. Pfadenhauer LM, Gerhardus A, Mozygemba K, Lysdahl KB, Booth A, Hofmann B, et al. Making sense of complexity in context and implementation: the Context and Implementation of Complex Interventions (CICI) framework. Implementation Science. 2017;12(1):21.
- 12. Fernandez ME, Walker TJ, Weiner BJ, Calo WA, Liang S, Risendal B, et al. Developing measures to assess constructs from the Inner Setting domain of the Consolidated Framework for Implementation Research. Implementation Science. 2018;13(1):52.
- 13. El-Jardali F, Lavis JN, Ataya N, Jamal D. Use of health systems and policy research evidence in the health policymaking in eastern Mediterranean countries: views and practices of researchers. Implement Sci. 2012;7:2.
