# Supplementary material for: Mixed method evaluation of the CEBHA+ integrated knowledge translation approach: a protocol
Source: Health Res Policy Syst. 2021 Jan 18;19:7. doi: 10.1186/s12961-020-00675-w (PMC7813167; doi:10.1186/s12961-020-00675-w)
Supplement: Supplementary file 2 — Additional file 2: CEBHA+ evaluation survey for researchers [file 12961_2020_675_MOESM2_ESM.pdf]

Thank you for your interest in taking part in this survey. Within the Collaboration for Evidence-based Healthcare and Public Health in Africa (CEBHA+), researchers and stakeholders collaborate on research projects to inform decision-making in healthcare and public health policy-and-practice. The purpose of this survey is to learn more about your experiences as a CEBHA+ partner in order to improve this engagement further - for both the researchers involved and the stakeholders from the policy-and-practice community.

This survey consists of 32 questions and statements about your engagement with CEBHA+. For each item, please respond by checking the option that best fits your experience and opinion. Please complete all questions. If a question does not apply, you can indicate this by checking the option "not applicable".

A "resume later" option is available if you want to pause the survey and save your answers.

We realise that the current situation may have a significant impact on both the content of your daily work, and current ways of working. Therefore, please refer to your experiences <u>before</u> the SARS-CoV-2 pandemic, when answering the questions.

If you have any questions, please contact the lead researcher: Kerstin Sell, MD MSc Pettenkofer School of Public Health LMU Munich email: ksell@ibe.med.uni-muenchen.de

Section A: Consent

A1. Your participation in this research is entirely voluntary. We will ask you to create an individual ID code to be used in this survey as well as in a subsequent survey in approximately 2.5 years. Apart from the individual ID code, your opinion and some demographic information, we will not ask you to provide any personal data such as your name or date of birth. Your information will be collected and analyzed in a pseudononymous form so it will not be possible for anyone to retrace what you have stated. This also applies to any reports or scientific publications resulting from this research. We will securely store collected data on an institutional storage provided by the LMU Munich in compliance with EU data protection regulations. We respect your trust and protect your privacy and will never share data with third parties.

I have read the above information and I consent voluntarily to be a participant in this survey. I agree with the collection and use of personal data and findings in accordance with the participant information. ☐

I do not want to participate in the study. ☐

Section B: Unique identification code

The following four questions are used to build a unique identification code that will help us to match your survey and interview data at different points in time. Please rest assured that your privacy is protected.

B1.

Instructions:

Insert two capital letters or two numbers per question, for example:

Birth month: if someone was born in March they should write "03".

First and last letter of one's first name: someone called Rosemary should write "RY".

First and last letter of the place of birth: if someone was born in Cape Town they should write "CN".

Write "DK" if you do not know the answer to the question.

Please answer the following:

Please indicate the month of your birth (numerical, i.e. 01-12)

B2.

Instructions:

Insert two capital letters or two numbers per question, for example:

Birth month: if someone was born in March they should write "03".

First and last letter of one's first name: someone called Rosemary should write "RY".

First and last letter of the place of birth: if someone was born in Cape Town they should write "CN".

Write "DK" if you do not know the answer to the question.

Please answer the following:

Please state the first and last letter of your own first name (not your initials)

B3.

Instructions:

Insert <u>two capital letters</u> or <u>two numbers</u> per question, for example:

Birth month: if someone was born in March they should write "03".

First and last letter of one's first name: someone called Rosemary should write "RY".

First and last letter of the place of birth: if someone was born in Cape Town they should write "CN".

Write "DK" if you do not know the answer to the question.

Please answer the following:

Please state the first and last letter of your place of birth (city or village)

B4.

Instructions:

Insert two capital letters or two numbers per question, for example:

Birth month: if someone was born in March they should write "03".

First and last letter of one's first name: someone called Rosemary should write "RY".

First and last letter of the place of birth: if someone was born in Cape Town they should write "CN".

Write "DK" if you do not know the answer to the question.

Please answer the following:

Please state the first and last letter of your mother's name

Section C: Demographic survey

C1. Which country do you represent in the CEBHA+ partnership?

- Ethiopia ☐
- Uganda ☐
- Rwanda ☐
- Malawi ☐
- South Africa ☐
- Other or multiple countries (please specify): ☐

Other or multiple countries (please specify):

C2. What is your age?

- 18 - 24 years ☐
- 25 - 34 years ☐
- 35 - 44 years ☐
- 45 - 54 years ☐
- 55 - 64 years ☐
- 65 years and over ☐

C3. What is your gender?

- Female ☐
- Male ☐
- I prefer not to say ☐
- Other gender: ☐

Other gender:

C4. What is the <u>highest degree</u> or level of schooling you have completed?

- Some high school, no degree ☐
- High school degree or equivalent ☐
- Some college credit, no degree ☐
- Trade/technical/vocational training ☐
- Associate degree (e.g. AA, AS) ☐
- Bachelor's degree (e.g. BA, BSc, BSN) ☐
- Master's degree (e.g. MA, MSc, MSN) ☐
- Professional degree (e.g. MD, DDS, DVM) ☐
- Doctorate degree (e.g. PhD, EdD) ☐
- Other (please specify) ☐

Other (please specify)

C5. **In which field did you receive this degree? (e.g. medicine, public health, political science, psychology, economics etc.)**

C6. **Which is the <u>main organisation</u> you represent in this partnership?**

- Academic university ☐
- Government department, agency or ministry ☐
- Regional or local health authority or equivalent ☐
- Research institute (not within a university) ☐
- Other (please specify) ☐

Other (please specify)

C7. **How long have you been employed in the <u>same department</u> within this organisation? (e.g. NCD department)**

- one year or less ☐
- 2 or 3 years ☐
- 4 or 5 years ☐
- 6 - 10 years ☐
- 11 - 15 years ☐
- more than 15 years ☐

C8. **How many years of professional experience do you have in your current field/discipline?**

- 5 years or less ☐
- 6 - 10 years ☐
- 11 - 15 years ☐
- more than 15 years ☐

C9. How long have you been actively engaged with CEBHA+?

- [ ] 0 - 6 months
- [ ] 6 - 12 months
- [ ] 13 - 24 months
- [ ] more than 24 months

C10. To your knowledge, have any research outputs been produced within the entire CEBHA+ research consortium (for example electronic newsletters, scientific publications, reports, issue briefs, plain-language summaries, conference presentations)?

- [ ] Yes
- [ ] Not yet

Section D: Frequency of Engagement

The following questions cover the frequency of your engagement with CEBHA+ partners from the policy-and-practice community.

Please refer to your experiences from before the SARS-CoV-2 pandemic, when answering the questions in this survey.

D1. Frequency of collaborative engagement

	Never	1 or 2 times	3 or 4 times	5 times or more	I don't know
How often have you met CEBHA+ partners from the policy-and-practice community in person (e.g. at conferences, workshops, local meetings etc.)?	☐	☐	☐	☐	☐

D2.

In which of the following ways have you been involved with CEBHA+ partners from the policy-and-practice community (=decisionmakers) and how often were you involved?

How often have you...

	Never	1 - 5 times	6 - 10 times	more than 10 times	I don't know
... communicated with CEBHA+ decision-makers on the phone? (including skype, zoom calls and GoToMeetings)	☐	☐	☐	☐	☐
... reached out to CEBHA+ decision-makers via email to discuss the ongoing research project?	☐	☐	☐	☐	☐
... been contacted by CEBHA+ decision-makers for some expert advice or referral to other experts?	☐	☐	☐	☐	☐
... interacted with CEBHA+ decision-makers on social media (twitter, facebook, instagram etc)?	☐	☐	☐	☐	☐
... interacted with CEBHA+ decision-makers using messenger services (whatsapp, threema, signal etc.)?	☐	☐	☐	☐	☐

	Never	1 - 5 times	6 - 10 times	more than 10 times	I don't know
... circulated (electronic) newsletters or other updates (via mail, print material etc.)?	☐	☐	☐	☐	☐

Section E: Individual characteristics

E1. **This section covers <u>your general perceptions</u> regarding the use of research evidence in the policy-and-practice community.**

Please refer to your experiences from before the SARS-CoV-2 pandemic.

Do you agree or disagree with the following statements?

	I agree	I disagree	I don't know/not applicable
Policy- and decision-makers show little regard for the value of evidence.	☐	☐	☐
Policy- and decision-makers have the expertise for acquiring, assessing quality and local applicability of research evidence, and applying it in health policy-making.	☐	☐	☐
Policy- and decision-makers do not use scientific evidence in the policymaking process even when it is provided to them.	☐	☐	☐
Policy- and decision-makers clearly articulate priorities for health systems and policy research.	☐	☐	☐
Policy formulation is usually based on internal discussions in the Ministry of Health, donor preferences, and impromptu processes rather than evidence-based processes.	☐	☐	☐
There is insufficient information about how health policies are being developed.	☐	☐	☐

E2. **The following questions cover your personal views regarding your engagement within the CEBHA+ project.**

On an <u>individual level</u>, do you agree or disagree with the following statements?

	I agree	I disagree	I don't know/not applicable
I have enough time to engage in the CEBHA+ research partnership.	☐	☐	☐
Based on my skills and knowledge, I feel confident in engaging in research partnerships like CEBHA+.	☐	☐	☐
I am committed to the collaborative engagement with decision-makers from the policy-and-practice community within CEBHA+.	☐	☐	☐
Engaging in mutually beneficial research partnerships to support decision-making makes sense to me.	☐	☐	☐
In research partnerships prior to CEBHA+ I have had predominantly good experiences.	☐	☐	☐

Section F: Context: barriers & facilitators

In the following section, we would like to hear about potential barriers and facilitators you have faced with respect to the collaborative engagement within CEBHA+ (that is the partnership component beyond your research work, project administration and planning etc).

Please refer to your experiences from before the SARS-CoV-2 pandemic.

F1. **Within your organisation, have you encountered any facilitators with respect to your engagement with CEBHA+ partners from the policy-and-practice community (i.e. factors that made it easier to engage with them)?**

If so, please indicate the three most relevant facilitators.

please check the three most relevant factors

- sufficient financial resources ☐
- adequate training/education ☐
- organisational/administrative or supervisor support ☐
- sufficient physical space (e.g. meeting rooms etc) ☐
- enough time to engage ☐
- incentives for engagement ☐
- a clear vision ☐
- motivation for the engagement ☐
- continuous staff involvement ☐
- other (please specify): ☐

other (please specify):

F2. **Within your organisation, have you encountered any barriers with respect to your engagement with CEBHA+ partners from the policy-and-practice community (i.e. factors that made it more difficult to engage with them)?**

If so, please indicate the three most relevant barriers.

please check the three most relevant factors

- lack of financial resources ☐
- lack of training/education ☐

lack of organisational/administrative or supervisor support ☐

lack of physical space ☐

lack of time ☐

lack of incentives ☐

lack of a clear vision ☐

lack of motivation ☐

lack of staff continuity ☐

other (please specify): ☐

other (please specify):

F3. Among the research and practice partners in the CEBHA+ project...

	Yes	No	I don't know
... roles and responsibilities are communicated explicitly and clearly among partners.	☐	☐	☐
... expectations regarding the research partnership are explicit and clear to all partners.	☐	☐	☐
... project goals and deliverables are explicit and clear to all partners.	☐	☐	☐

F4. The research findings from CEBHA+ were presented to stakeholders...

	Yes	No	I don't know
...in a format suitable for policy-and/or-practice partners.	☐	☐	☐
...in a language suitable for policy-and/or-practice partners.	☐	☐	☐
...in a timely manner.	☐	☐	☐

Section G: Capacity building

G1. Please refer to your experiences from before the SARS-CoV-2 pandemic, when answering the questions.

Being part of this research partnership...

Please check the answer option that fits your experience and opinion best.

	not at all	not really	undecided	somewhat	very much
...improved my access to up-to-date information relevant to my research.	☐	☐	☐	☐	☐
…improved my access to contacts who I reach out to to obtain information that is relevant to my work.	☐	☐	☐	☐	☐
...increased my personal knowledge or understanding about the health issues addressed in CEBHA+.	☐	☐	☐	☐	☐
...changed my beliefs/understanding with respect to an intervention, a topic, or a group of people.	☐	☐	☐	☐	☐
...confirmed my views about the importance of the health issues addressed.	☐	☐	☐	☐	☐
...increased my confidence in my professional practice or day-to-day activities.	☐	☐	☐	☐	☐

G2. Being part of this partnership in CEBHA+...

Please check the answer option that fits your experience and opinion best.

	not at all	not really	undecided	somewhat	very much
...helped increase my skills in developing research questions.	☐	☐	☐	☐	☐
...increased my receptiveness to new ideas or evidence.	☐	☐	☐	☐	☐
...improved my ability to know how to find or access relevant research information.	☐	☐	☐	☐	☐
...enhanced my personal ability or confidence to conduct a research activity.	☐	☐	☐	☐	☐
...provided me with an opportunity for professional or personal development (e.g. building research skills or enhancing statistics skills etc).	☐	☐	☐	☐	☐
...led me to pursue different activities to develop my research skills (e.g. attending a course).	☐	☐	☐	☐	☐

G3. As a result of my partnership with CEBHA+, I feel comfortable taking part in similar engagement activities in the future.

(e.g. partner with stakeholders to interpret research findings, engage in new research partnerships)

- I agree ☐
- I disagree ☐
- I don't know/not applicable ☐

Section H: Relationship building

H1. **This section covers the relationship between researchers and their partners from the policy-and-practice community.**

Please refer to your experiences from before the SARS-CoV-2 pandemic.

Within CEBHA+...

Please check all that apply

...a common language is used by all parties (i.e. terminology, common definitions etc).	☐
...partners make their needs and constraints explicit.	☐
...communication is frequent.	☐
...I am able to express my views freely.	☐
...I feel that my views are heard.	☐
...the respective organisational realities of research partners are being discussed.	☐
...the collaborating partners trust each other.	☐

Section I: Collaborative research

I1. **The following questions cover the degree of collaboration between researchers and partners from the policy-and-practice community.**

Please refer to your experiences from before the SARS-CoV-2 pandemic.

Within the CEBHA+ research partnership, partners from research and practice in your country jointly...

If the research project did not reach the respective stage yet, please check 'not applicable'.

	Yes	No	Not applicable
...identified the research questions.	☐	☐	☐
...designed the research protocols.	☐	☐	☐
...conducted data collection.	☐	☐	☐
...analysed data.	☐	☐	☐
...evaluated the relevance of research (e.g. of the current project, of new findings etc).	☐	☐	☐
...discussed the findings and implications.	☐	☐	☐

I2. Within the CEBHA+ partnership, do you agree or disagree with the following statements?

	I agree	I disagree	I don't know/not applicable
The individuals involved represent a broad range of perspectives.	☐	☐	☐
CEBHA+ partners in the policy-and-practice community value my contributions.	☐	☐	☐
All partners, i.e. CEBHA+ researchers and actively involved stakeholders, are acknowledged in CEBHA+ project documents (e.g. reports, posters, publications).	☐	☐	☐
The collaboration between stakeholders and researchers in CEBHA+ added value to the ongoing research.	☐	☐	☐

Section J: Intermediate outcomes

J1. This section covers outcomes of the CEBHA+ partnership.

Do you agree or disagree with the following statements?

Please refer to your experiences from before the SARS-CoV-2 pandemic.

The research evidence produced in CEBHA+...

	I agree	I disagree	I don't know/not applicable
... is relevant to the health issues addressed by the partnership.	☐	☐	☐
... is directly applicable.	☐	☐	☐
... is trustworthy.	☐	☐	☐

Section K: Uptake

K1. The last question is on the broader impact of your engagement in CEBHA+.

Please refer to your experiences from before the SARS-CoV-2 pandemic.

From my experience, compared to research projects <u>without a policy-and-practice partnership</u>, the research evidence produced in CEBHA+ is...

	I agree	I disagree	I don't know/not applicable
...more likely to be used in healthcare/public health decision-making.	☐	☐	☐
...more likely to have a lasting impact on public health.	☐	☐	☐

K2. **Do you have any other thoughts or comments with respect to your engagement in CEBHA+ that you would like to share?**

How has your engagement with CEBHA+ stakeholders changed during the SARS-CoV-2 pandemic? Have you had to pause the engagement or have you been working with some stakeholders on pandemic-related work?

Thank you for your participation!

If you have any questions, please contact the lead researcher:

Kerstin Sell, MD MSc Pettenkofer School of Public Health LMU Munich

email: ksell@ibe.med.uni-muenchen.de
