# Supplementary material for: Mixed method evaluation of the CEBHA+ integrated knowledge translation approach: a protocol
Source: Health Res Policy Syst. 2021 Jan 18;19:7. doi: 10.1186/s12961-020-00675-w (PMC7813167; doi:10.1186/s12961-020-00675-w)
Supplement: Supplementary file 4 — Additional file 4: CEBHA+ evaluation interview guide for researchers [file 12961_2020_675_MOESM4_ESM.docx]

**Interview Guide for CEBHA+ Researchers**

**General information**

| Name of the interviewer |  |
| --- | --- |
| Name of the research assistant/note taker |  |
| Date of the interview [dd.mm.yyyy] |  |
| Location |  |
| Country |  |
| 8-digit ID of interviewee (self-generated): |  |
| - Month of birth (numerical, i.e. 01-12) |  |
| - First and last letter of interviewee’s first name |  |
| - First and last letter of interviewee’s place of birth |  |
| - First and last letter of interviewee’s mother’s name |  |
| Language spoken |  |
| Start point of the interview [hh:mm] | ____:____ |
| End point of the interview [hh:mm] | ____:____ |

**Interviewer instructions**

- Welcome the interview partner and introduce yourself by name and function.
- Create a warm and easy-going atmosphere.
- Thank the interview partner for taking the time to take part in this research. Explain the purpose and the topic of the interview
  - Overall objective is to find out whether and how this research partnership in CEBHA+ contributes to an increased uptake of research evidence in policy and practice decision-making. Thus the focus of the interview is on the partnership, not the ongoing research activities.
  - In particular, we want to know
    - How does this partnership work?
    - What are the results of this partnership?
- Explain what will happen during the interview.
- Explain that ethical approval for this study was granted locally and at the German partner university LMU.
- Go through the informed consent form and have the interviewee sign the statement. Ask the interviewee if there are any questions before starting the interview.
- Explain why the interview will be tape recorded and ask for permission.
- Start the tape.

| **#** | **Main topic** | **Probing questions** | **Intention of question** | **Corresponding survey constructs/items** |
| --- | --- | --- | --- | --- |
| 0 | Researcher’s background | - Ask the researcher to describe where he/she works and what his/her professional background is - Previous work experience with stakeholder engagement? |  |  |
| 1 | To start our conversation, would you kindly tell me about the partnership between you and the CEBHA+ policy or practice partners? | - Who are the decision-makers you are engaging with within CEBHA+? [aim to discuss questions 1-3 with respect to every decision-maker] - When was the partnership with this partner initiated? - Who initiated the partnership? - Why did they initiate the partnership? - How and how often do you interact with [insert CEBHA+ partner’s name]? - Has this partnership been formalised (e.g. by a memorandum of agreement) or is it a rather informal agreement/contact? - Have you ever been in a similar partnership? - If yes: How does this partnership differ from other partnerships? | - To find out about the beginning of the partnership. - To find out about the formalisation or institutionalisation of this partnership - To find out about previous partnerships | Implementation process, initiation |
| 2 | Let us talk about the research activities you are involved in. | - What research activities do you work on with [insert CEBHA+ policy or practice partner]? - What is your role in the [planned] research activities in CEBHA+? - How were roles, goals and respective expectations regarding this research task communicated with the policy and practice partner? - How would you describe the collaboration with the policy and practice partner on these research activities? - So far, at which stages of the research process have you collaborated closely with the partner? (development of the research question, stakeholder consultation, implementation, other?) | - To find out about research activities and collaboration | Collaborative Research |
| 3 | Now, I would like to talk a bit more about your personal relationship with this partner from policy-and-practice. | - What characterises your partnership?   [Probe for mutual trust, understanding, respect, professional, distanced, warmth…]   - How do you feel that your personality, identity, position, and professional background impacts on the relationship? | - To learn about the relationship between the CEBHA+ researcher and decision-maker & characteristics of the partnership | Relationship-building |
| 4 | NEW | - How has your engagement with CEBHA+ stakeholders changed during the SARS-CoV-2 pandemic? - Have you had to pause the engagement or have you been working with some (or new) stakeholders on pandemic-related work? | - to find out whether the partnership was mainted during the pandemic and how it may have evolved |  |
|  | **If time permits, go through questions 1 – 3 with respect to the other collaboration partners.** | | | |
| 5 | Next, I would like to talk about your expectations regarding the research partnership, its potential results and long-time impact. | **Expectations**   - What do or did you think could be **benefits** of this partnership? - Have you encountered any of them so far? - Are there expectations that have not been met so far? Why? - Do or did you think there could be **negative or unintended consequences** of this partnership? - If yes, have you encountered any so far?   **Relevance/applicability of results**   - What do you think about the scientific relevance of CEBHA+ results? - What do you think about the applicability of CEBHA+ results in your local context?   **Impact**   - What do you think will be the long-time impact of the research (i.e. scientific findings) conducted in the course of this partnership?   **Improvement**   - How could the partnership be improved to increase the value and impact of the research evidence produced in CEBHA+? - Probe for training needs (lack of knowledge/skills) | - To find out about expectations regarding the partnership - To find out about the potential outcomes and impact of the partnership - Potential for improvement | Intermediate outcomes and impact of the partnership |
| 6 | Let us talk about a few other aspects regarding the CEBHA+ project. | - How do you perceive the **complexity** of the research topic? - How do you perceive the complexity of the methods and research activities pursued in this partnership? - How do you feel the **cross-country exchange** within the CEBHA+ IKT group influences your local engagement with decision-makers? - How do you feel does the **evaluation** influence IKT engagement? | - To investigate perceptions of complexity - Role of the cross-country network - Role of the evaluation | Project-specific context |
| 7 | How does the context here in [insert country of interviewee] influence the research partnership you are in? | - Are there any contextual barriers which could potentially affect this partnership? - Are there any contextual facilitators which influence this partnership? (that is factors that make it easier to engage in a research partnership such as CEBHA+?)   [Probe for geographical e.g. distance between researcher and stakeholder, legal, socio-economic, cultural, political, ethical and epidemiological context] | - Influence of macro context on the partnership | Macro context |
| 8 | Before wrapping up, I would be interested in your very personal views on some aspects within the CEBHA+ partnership. | - What does the partnership with decision-makers/ collaborative aspect of CEBHA+ mean to you? - How is it relevant for your research work? - Is this collaboration considered important within your organisation? Is it supported? Are new ideas with respect to IKT taken up? | - Understanding and value of IKT - institutional absorptive capacity |  |
| 9 | “ | - For someone starting a similar research partnership, what would be your advice to them? | Appreciation of participant’s expertise and opinion |  |
| 10 | Is there anything else you wish to add at this point? | Are there any other thoughts you’ve had during your involvement within CEBHA+ that you would like to share? Any comments? |  |  |
| 11 |  | Finally, would you like to receive a copy of the interview transcript to review the content before we start analysing the interview data? If so, please provide your email address. |  |  |

**Adaptations/focus for PIs, coordinators:**

- professional background, length of involvement in CEBHA+
- role in the development of the CEBHA+ approach, including IKT approach
- participation in any stakeholder engagement
- expectations regarding IKT, observed effects thus far
- recommendations to improve current IKT approach, IKT in future collaborations
